# Effect of altitude on COVID-19 mortality in Ecuador: an ecological study

**DOI:** 10.1186/s12889-021-12162-0

**Published:** 2021-11-12

**Authors:** Adriana Campos, Bridget Scheveck, Jeegan Parikh, Santiago Hernandez-Bojorge, Enrique Terán, Ricardo Izurieta

**Affiliations:** 1 University of South Florida, 4202 E Fowler Ave, Tampa, FL 33620 U.S.; 2 Colegio de Ciencias de la Salud, Universidad San Francisco de Quito, Campus Cumbayá, Diego de Robles s/n, Quito, 170901 Quito, Ecuador

**Keywords:** SARS-CoV-2, COVID-19, Epidemiological study, Mortality rates, Ecological factor, Altitude

## Abstract

**Background:**

The SARS-CoV-2/COVID-19 pandemic has claimed nearly 900,000 lives worldwide and infected more than 27 million people. Researchers worldwide are studying ways to decrease SARS-CoV-2 transmission and COVID-19 related deaths. Several studies found altitude having a negative association with both COVID-19 incidence and deaths. Ecuadorian data was used to explore the relationship between altitude and COVID-19.

**Methods:**

This is an ecological study examining province-level data. To explore a relationship between altitude and COVID-19, this study utilized publicly available COVID-19 data and population statistics. ANOVA, correlation statistics, and a multivariate linear model explored the relationship between different Ecuadorian altitudes against incidence, mortality, and case-fatality rates. Population statistics attributed to COVID-19 were included in the linear model to control for confounding factors.

**Results:**

Statistically significant differences were observed in the regions of Amazónica, Sierra, Costa of Ecuador for incidence, mortality, and case fatality rates, suggesting an association between altitude and SARS-CoV-2 transmission and COVID-19 disease severity (*p*-value ≤0.05). In univariate analysis, altitude had a negative association to mortality rate with a 1-unit change in altitude resulting in the decrease of 0.006 units in mortality rate (*p*-value = 0.03). The multiple linear models adjusted for population statistics showed a statistically significant negative association of altitude with mortality rate (*p*-value = 0.01) with a 1-unit change in altitude resulting in the decrease in mortality rate by 0.015 units. Overall, the model helped in explaining 50% (R^2^ = 0.4962) of the variance in mortality rate.

**Conclusion:**

Altitude may have an effect on COVID-19 mortality rates. However, based on our model and R^2^ value, the relationship between our variables of interest and COVID-19 mortality may be nonlinear. More research is needed to understand why altitude may have a protective effect against COVID-19 mortality and how this may be applicable in a clinical setting.

## Background

SARS-CoV-2 was identified in China towards the end of 2019 when a cluster of acute respiratory tract infections occurred in the Hubei providence. During the initial outbreak, the virus was discovered to cause an acute respiratory syndrome, named coronavirus disease 2019 (COVID-19), mostly affecting the elderly (i.e., over 60 years of age) and individuals with underlying health conditions [1, 2]. However, the viral mode of transmission and globalization allowed this regional outbreak to spread throughout the world. By the spring of 2020, SARS-CoV-2 was present in every continent excluding Antarctica [3]. At the time this article was written, SARS-CoV-2 has infected more than 27 million individuals worldwide and has caused nearly 900,000 deaths [4]. From viral transmission mechanisms [5–8] to COVID-19 treatments [9], researchers are attempting to better understand the disease dynamics and reduce its burden on mankind. The global spread of COVID-19 cases drives research to attempt to understand how environmental factors may contribute to SARS-CoV-2 transmission and COVID-19 disease severity [5, 7, 10–14].

One ecological factor suggesting an effect on viral transmission and disease severity is altitude [14–17]. There is growing body of evidence indicating altitude may have an effect on disease incidence [17, 18] as well as overall viral integrity [14–16, 19, 20]. One study in particular illustrated a distinct decrease in COVID-19 affected populations above 3000 m above sea level (masl) [14]. Furthermore, an Indian study found a similar conclusion [16]. While the two aforementioned studies focused on disease incidence and altitude, there are also studies examining viral properties that may be affected by higher altitudes [15, 20]. This associated decrease may be due to biological factors in susceptible humans, environmental factors affecting SARS-CoV-2 and its transmission dynamics, or perhaps a combination of both. Moreover, these studies propose a negative correlation between altitude and disease incidence.

There are few studies investigating the effect altitude may have specifically on COVID-19 mortality [21, 22]. Of the two studies found, both offer different conclusions, indicating a need for additional research. One of the studies, conducted in Peru, found high altitude reduced SARS-CoV-2 infection rate, yet the case fatality rate (CFR) was not affected by altitude [21]. However, a letter to the editor addressing the aforementioned study suggests their conclusion may be due to differences in regional testing rates [23]. Another study examining the CFRs in United States and Mexican residents living at different altitudes concluded the CFR was only statistically lower in men younger than 65 years of age [22]. It is evident additional research is required to further explore the relationship between altitude and COVID-19 mortality.

**Fig. 1:**
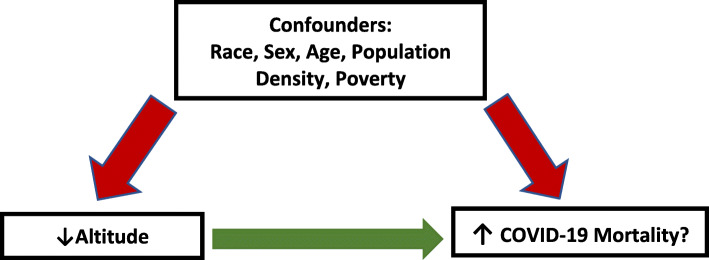
Operationalization of the research question in relation to the covariates. In this figure we show the relationship the potential confounders have on altitude and COVID-19 mortality

During the early stages of the COVID-19 pandemic, Ecuador became one of the hardest hit South American countries, having one of the highest case fatality rates in the world [24]. Its first case, confirmed in February 2020, appeared in its largest city, Guayaquil, and shortly thereafter cases started appearing in the rest of the Ecuadorian provinces [25–27]. By the end of August 2020, nearly 100,000 cases and 10,000 deaths were confirmed [28]. Ecuador’s high caseload and large altitudinal range provide the perfect setting to explore communities living at different altitudes during this pandemic. This study aims to explore how COVID-19 mortality statistics may be affected by altitude (Fig. 1).

## Methods

### Study setting and design

Ecuador, the setting of our study, contains numerous ecological regions ranging from Galapagos Islands to the Amazónica. The provinces are categorized into four regions of Amazónica, Sierra [Highlands], Costa, and Insular [Galapagos Islands]. In addition to its ecological diversity, Ecuador is home to populations living in areas ranging from sea-level to 3000+ meters in altitude. Ecuadorian regions are mostly divided on altitude/ecological level, with provinces categorized as “Costa” falling between 50 to 1000 masl, “Sierra” 800 to 3900 masl, “Amazónica” 500 to 1500 masl, and “Insular” masl.

This is an ecological study, conducted using Ecuadorian province-level data on COVID-19 case counts and deaths from February 2020 until August 2020. All the provinces (*n* = 23), except for the Galapagos Islands (“Insular”) were included in the study.

### Study data

All the data used in the study was publicly available and extracted manually from the Ecuadorian Ministry of Public Health COVID-19 bulletin [MOH] and Ecuadorian National Institute for Statistics and Censuses [INEC]. Information relating to COVID-19, such as the number of confirmed cases and deaths per provinces was extracted from the MOH monthly COVID-19 bulletin [28]. All the variables showing relevance to COVID-19 transmission based on current literature and being available at provincial level were included in the study. Due to last census taking place in 2010, the INEC census estimates for 2020 were used. Statistics on poverty, sex, population density, age groups, race, and hospital beds per 1000 persons were taken from INEC database [29]. The estimates of each of the sociodemographic covariates were calculated by using 2010 covariate proportions on 2020 INEC census estimates. The healthcare resource availability proxy (i.e., hospital beds per 1000 persons) was directly from INEC 2020 data. As wide ranges of altitudes are possible for each of the provinces, the altitude of each province’s capital was used, assuming a significant portion of each province’s population would be in the capital as well as most of the province hospitals. All the provincial-level data were collated and input in a single excel file. None of the variables from the two data sources had any missing variables.

### Statistical analysis

From the COVID-19 case and death counts data incidence, mortality, and case-fatality rates were calculated on the provincial level. We initially computed basic descriptive statistics on regions with provinces as unit of analysis. Due to natural categorization of provinces, a high degree of collinearity existed between region and altitudes of provinces. An ANOVA conducted comparing altitude to region further confirmed this assumption with all regions being statistically different and therefore allowing the use of regions as a valid proxy for altitude for the ANOVA. The ANOVA procedure was used to compare the regional differences in incidence, mortality, and case-fatality rates. It was also run to compare the regional differences in independent variables and other covariates. Due to Insular region having just one province, to determine a better fit, an ANOVA procedure was done with and without its inclusion and therefore, the subsequent analyses excluded this region. Univariate procedures to examine the incidence, mortality, and CFRs distributions were conducted. Due to the covariate data being related specifically to the population and not to outcome, CFR was not deemed appropriate to create exploratory models, and therefore, the model outcome of interest was mortality rate.

Initially, correlation statistics was conducted on all covariates against mortality rate. Based on the results of the regional ANOVA analysis and the correlation statistics, a multilinear modeling was conducted. The model aimed to explore if altitude could explain the differences in regional mortality rates. As altitude was the main area of interest, we excluded region in the final model to avoid instability created by the high intercorrelation between region and altitude. The initial regression model included sociodemographic variables. To account for healthcare resource availability the hospital beds per 1000 persons 2020 estimates were added to the initial model. Based on the results of the sequential regression models, the final model controlled for population density, proportion of males, level of poverty, proportion of people above the age of 55 years, and race. Covariates listed were included due to its significance in the literature as potential confounders for COVID-19 mortality, regardless of the level of statistical significance in either correlation statistics or model. We then assessed homoscedasticity and the distribution of the residuals to determine if the assumptions of multiple linear regression were met. Statistical significance was defined as having a *p*-value less than or equal to 0.05. All the data was analyzed using SAS® 9.4 software [30].

## Results

### Basic descriptive statistics and ANOVA

Twenty-three Ecuadorian provinces in three regions were analyzed (*n* = 11 Sierra, *n* = 6 Costa, n = 6 Amazónica). Table 1 indicates the regional differences found in the independent variables. Altitude and the population over 55 years old were the most statistically different between regions (*p*-value < 0.0001), while poverty, sex, population density, and race were significant at p-value < 0.05. Hospital beds per 1000 persons was not statistically significant.

**Table 1:** Regional wise differences in independent variables

Covariates	Costa	Amazónica	Sierra
Altitude (masl) **	21	694.17	2408.18
Poverty (%) *	71.07	78.28	61.06
Male (%) *	51.09	51.89	44.86
Population above 55 years of age (%) **	12	8.69	14.58
Population density*	106.17	6.50	94.64
Race (%) *			
Indigenous	1.15	34.27	14.14
Afro Ecuadorian	13.53	2.76	3.31
Montubio	12.61	0.60	0.92
Mestizo	65.91	58.79	77.67
White	6.1	3.29	3.79
Other	0.67	0.28	0.16
Hospital beds per 1000 persons	1.15	1.20	1.35

*p-value < 0.05 ** p-value < 0.0001 Measured by F-test (ANOVA)

**Fig. 2:**
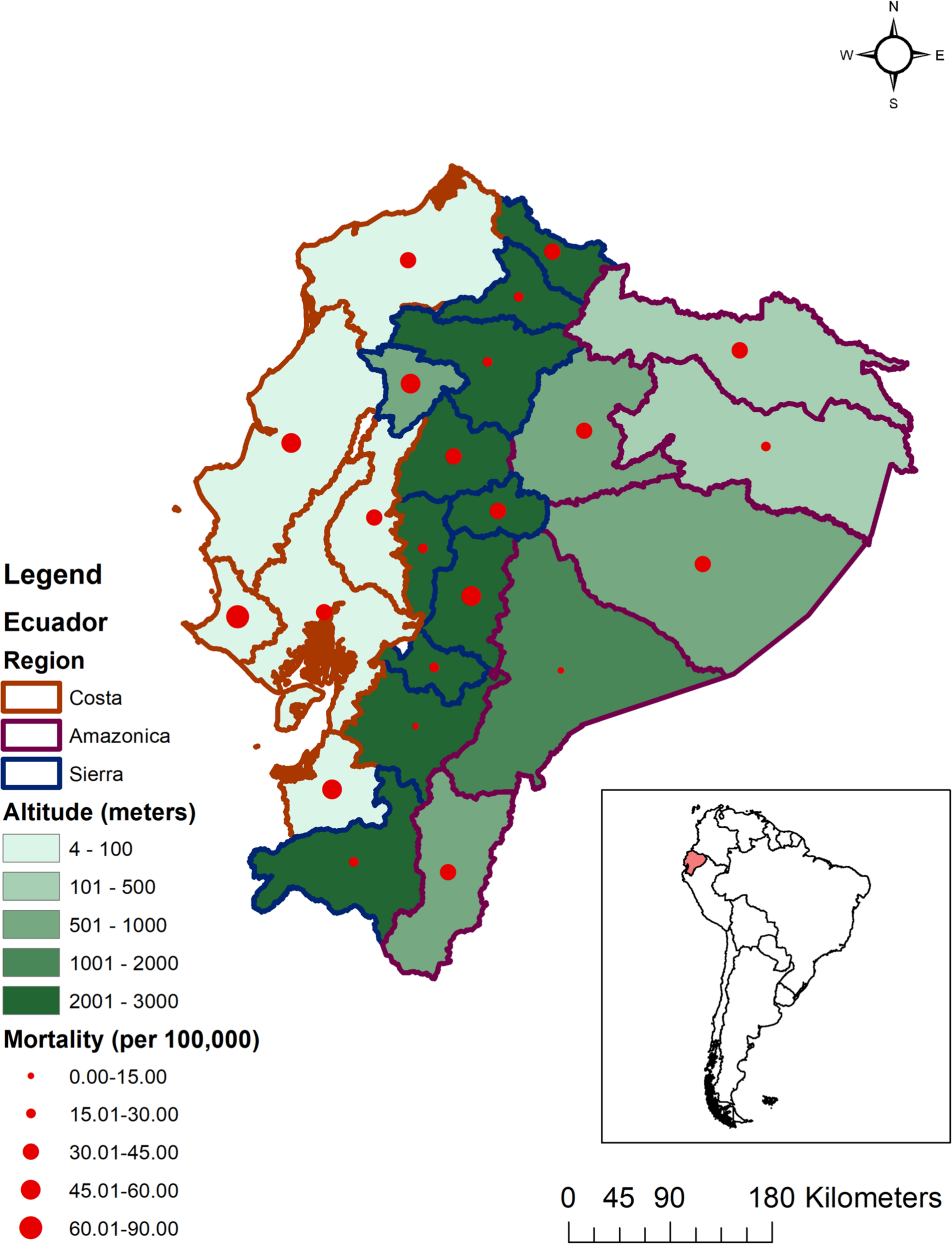
Provincial COVID-19 mortality rates and altitudes, Ecuador. In this map of Ecuador, the provinces with brown borders represent Costa region, blue borders Sierra region, and purple borders Amazónica region. The provinces are divided in five categories based on the altitude of the capital of those provinces in meters and are represented in light to dark green. Mortality rates due to COVID-19 in each of the provinces were categorized into five levels (0–15, 15–30, 30–45, 45–60, 60–90) and are presented with ascending size of red dots. The right lower corner shows a map of South America with Ecuador highlighted in red

Regional differences were observed for mortality (Fig. 2), case-fatality, and incidence rates (Table 2). The Amazónica mortality rate mean was 29.96 per 100,000, the Costa mean was 49.46 per 100,000, and the Sierra regional mean was 30.96 per 100,000. The ANOVA result for mortality rates between regions was statistically significant (*p*-value = 0.05, F-test = 3.44, df = 22). None of the individual regional comparisons were statistically significant (e.g., Sierra versus Costa, etc.). The CFR means for Amazónica, Costa, and Sierra were 2.97, 11.94, and 5.19 per 100 cases respectively. The regional CFR was significantly different (*p*-value = 0.01, F-test = 6.71, df = 22) and there were statistically significant region comparisons by Costa vs. Sierra and Costa vs. Amazónica (*p*-value < 0.05). The mean incidence rates for Amazónica, Costa, and Sierra were 1074.86, 461.16, and 628.32 per 100,000, respectively. The regional incidence rates were significantly different (*p*-value < 0.0001, F-test = 18.64, df = 22), with Amazónica having a significantly higher incidence rate compared to both Costa and Sierra (*p*-value < 0.05).

**Table 2:** Regional differences in mortality, case fatality, and incidence rate

Indicator	Costa	Amazónica	Sierra
Mortality rate**	49.45	29.96	30.96
Case Fatality rate*	11.94	2.97	5.19
Incidence rate*	461.16	1074.86	628.32

*p-value < 0.05 ** *p*-value = 0.05 Measured by F-test (ANOVA)

Note: Mortality rate and Incidence rate are calculated per 100,000 population, while Case Fatality rate is calculated per 100 cases

### Correlations

Correlation statistics between the covariates and the mortality rate were ran to explore any association between both independent and dependent variables that may be important to note in the final linear model. Altitude and Race (Other) were the only two statistically significant correlations to the mortality rate. Altitude showed a negative correlation of − 0.45 (*p*-value = 0.03) and Race (Other) of 0.61 (p-value = 0.002). All other covariates showed small correlations against mortality rate.

### Linear model

When adding healthcare resource availability to the initial model, the difference between R^2^, 0.002, was not statistically significant, therefore excluded from the final model. The final linear model included population density, proportion of males, poverty level, proportion above 55 years of age, altitude, and Race (White) (Table 3). The model was nonsignificant with a *p*-value of 0.06 and explained 50% of the variance in mortality rate. Individually, altitude was statistically significant with a p-value of 0.01. Population density was the next closest to significance at p-value = 0.06. No other risk factors were statistically significant.

$$ Mortality\ Rate=61.37+0.17\ \left( population\ density\right)-0.78\ (male)+0.29\ (poverty) $$

$$ -0.02\ (altitude)+1.76\ \left( above\ 55 yrs\right)-520.52\ (white) $$

**Table 3:** Univariate and Multivariate co-relations of risk factors with mortality rate

Risk factor	Univariate Estimates	Multivariate Estimates
Altitude	−0.006*	− 0.015*
Poverty	0.319	0.293
Male	−0.047	−0.775
Population density	0.031	0.170**
Population > 55 years	−0.632	1.756
White	144.087	−520.517***

*p-value < 0.05 **p-value =0.06 ***p-value = 0.09 Measure by F-test (ANOVA)

## Discussion

The results from the linear regression analysis clearly indicates altitude as the most statistically significant model variable. This was both the case while analyzing the variable at the univariate and multivariate level. Furthermore, the results from the ANOVA regional analysis, where regions were proxy for different altitudes, further showed a difference between incidence, case-fatality, and mortality rates. Amazónica had the highest incidence yet the lowest case-fatality and mortality rates. Compared to the other regions, Amazónica had the highest poverty level and proportion of males which are believed to be positively associated with higher COVID-19 morbidity and mortality [31, 32] and lowest population density, which is associated with lower incidence [31], potentially contradicting our observations. The high incidence rate in the Amazónica region may be explained by case misclassification with reports showing concern for potential dengue cases being diagnosed as COVID-19 [33]. Specifically in Ecuador, dengue misclassification and underreporting is a current concern during the COVID-19 pandemic [34]. This could explain the significantly higher incidence rate in the Amazónica, yet lowest morality statistics. Furthermore, it is well known due to the lack of sufficient health care facilities in the Amazónica, most morbidity indicators are underreported. However, in this analysis the healthcare resource availability proxy used did not convey statistically significant differences between regions. This may be due to lack of statistical power or the underrepresentation of hospital beds during the COVID-19 pandemic in Ecuador, where there were many provisional beds made for SARS-CoV-2 infected individuals. Conversely, the Costa region held the lowest incidence rate and highest case-fatality and mortality rate. Although the Costa region’s mortality rate was not statistically different than the Sierra region, the case-fatality rate was significantly higher. Since case-fatality rate is an indicator based on the number of deaths and the number of cases, not taking into account the population, it is important to acknowledge the differences in the number of case deaths varying from region to region. The high CFR in the Costa region may be due to a higher population density than both other regions. For both mortality and case-fatality rates, as the population-density increased so did mortality. The Costa region is home to the most populated Ecuadorian city and filled with both urban and rural communities. This region is then followed by the Sierra, with a similar mix of communities, while the Amazónica region is almost exclusively rural, with very few large cities in the region. However, one of the strongest risk factors found in the literature for COVID-19 mortality, advanced age [2, 32], was significantly the highest in the Sierra region. This could indicate not only altitude differences but also resource-oriented healthcare factors which could explain the higher mortality statistics in the Costa region [35, 36]. However, our chosen proxy for healthcare resource availability failed to demonstrate a difference between regions.

In both the correlation analysis and the linear model, altitude was a protective factor against COVID-19 mortality. Based on standardized estimates, altitude was the factor with the highest effect on mortality rates followed by population density and identifying as White. The rest of the covariates in the model were not statistically significant nor had large effect on mortality rate. While, race (Other) was statistically significant in the correlation analysis, the proportion of the population identifying as “Other” was too small to be clinically relevant. White race fit the model best and therefore included in the model. The healthcare resource availability covariate was nonsignificant in both ANOVA and linear regression models. Deemed COVID-19 risk factors, the covariates lack of significance may be due to lack of statistical power.

Several hypotheses have been proposed as possible explanations for the protective nature of altitude. One hypothesis focuses on the effects of chronic hypoxia in high altitude environments [37]. Specifically, examining the effects of chronic hypoxia on Angiotensin-Converting Enzyme 2 (ACE2), the enzyme SARS-CoV-2 binds to enter host cells. Under conditions of chronic hypoxia, Angiotensin-Converting Enzyme 1 (ACE1) is upregulated by Hypoxia Inducible Factor 1 (HIF-1) in human pulmonary artery smooth muscle cells shifting the balance of the oxygen sensitive Renin–Angiotensin System (RAS) away from the vasodilator ACE2 and towards the vasoconstrictor ACE1. This process markedly decreases ACE2 expression in the pulmonary artery smooth muscle cells [17, 18]. Due to SARS-CoV-2 utilization of the ACE2 receptor for cellular entry, it is hypothesized populations living in constant hypoxia may be less susceptible to SARS-CoV-2 infection. Additionally, HIF-1, activated in chronic hypoxia, may ameliorate a COVID-19 infection [38]. It is also possible that the levels of hypoxia encountered by populations in higher altitudes may have elicited adaptations with the potential to resist SARS-CoV-2 related complications. The hypoxemia may optimize cellular oxygenation, antioxidant systems and mitochondrial performance at the alveolar level [39]. Lastly, high concentrations of erythropoietin (EPO) might protect high altitude residents by stimulating erythropoiesis and heme synthesis and thereby increasing tissue oxygenation [40]. In addition to biological mechanisms that may be affected by living in higher altitudes, environmental factors are also theorized reasons for the observed negative correlation between altitude and COVID-19 mortality. A possible explanation of decreased SARS-CoV-2 infection rates in populations at high altitude is a higher level of O_3_ (ozone). Considered a disinfecting agent, ozone disrupts the reproductive cycle via peroxidation, affecting virus-to-cell contact and damaging the viral capsid [15, 41]. Further suggesting the potential impact ozone may have on SARS-CoV-2 transmission, one study, conducted in China from January to March 2020, showed a statistically significant negative correlation between ambient average ozone levels and number of confirmed cases [42]. Lastly, another common element found in high altitudes an increase of ultraviolet (UV) radiation. UV radiation was found to effectively eliminate SARS-CoV [43], suggesting a similar effect for SARS-CoV-2. However, studies on this hypothesis are limited and a recent study found no association between UV radiation and COVID-19 cases [44]. In addition to the sterilization effect of UV radiation, UV radiation’s effect on Vitamin D production is also an area of interest. Vitamin D is theorized to help maintain a healthy immune system and its deficiency is associated with increased risk of respiratory infectious diseases [45–48]. This is further supported by recent studies examining finding a protective effect of Vitamin D_3_ against COVID-19 [49, 50].

Studies have demonstrated that high altitudes are correlated with lower air density and greater distance between molecules which may reduce the size of the airborne virus inoculum and the probabilities of dissemination between people [14]. Furthermore, there may be behavioral factors affecting the relationship between COVID-19 mortality and altitude. Numerous other socioeconomic and lifestyle factors could explain the differences seen in regard to the infectivity and mortality in high altitude populations. A recent study found that higher altitude (> 2133 masl) might attenuate infection and death in counties of the United States of America. While infection rate did not correlate with population density, deaths per 100,000 showed a positive association with population density at higher altitudes and lower altitudes [51]. Arias-Reyes et al. concluded the incidence of COVID-19 decreases significantly starting at 1000 masl in 23 Latin American countries. The epidemiological model used in this study (SEIR) supported the hypothesis of decreased SARS-CoV-2 virulence in highlands in four South American countries: Argentina, Bolivia, Ecuador, and Peru [52]. There were higher percentages of recovered patients from the highlands when compared to lowlands, suggesting a higher recovery rate in South American territories with higher altitudes (> 1000 masl) [52]. Similarly, a study conducted in Brazil, concluded the COVID-19 relative incidence, the COVID-19 relative death rate, and the air relative humidity (RH) were lower in cities located in higher altitudes (795 < altitude ≤1135 masl) compared to other cities with middle (97 < altitude ≤795 masl) and low altitudes (altitude ≤97 masl) [53]. Additionally, Thomson et al. provided evidence of a protective effect of altitude from COVID-19 mortality in populations located in cities with 2500 masl. This protection was shown to be independent of poverty levels among the Peruvian population and inversely correlated with the prevalence of hypertension and hypercholesterolemia [54]. Conversely, results of Arias-Reyes et al. did not show significant difference of death-to-case ratio (death/total reported cases) between < 1000 masl areas when compared to > 1000 masl areas [52]. Another publication cautioned the researchers against associating altitude to decreased COVID-19 pathogenicity based solely on altitude arguing factors such as population density, access to commodities, clinical care, and ability to “social distance” may all be contributing to the observed reduced pathogenicity [55]. However, the results of our study suggest altitude is an independent protective factor even after considering population density or poverty. Health benefits of communities living in high altitude is not new knowledge, with high altitude associated with higher levels of physical activity, lower rates of obesity, cardiovascular disease, and cancer [56–58].

Limitations in the study mainly include lack of access to smaller unit-sized data and individual data. This study is an ecological study, focusing on province-level data, and should be noted that there may be individual cities that may be influential or leveraging the results. Regardless of the limitations, altitude was significantly protective against COVID-19 mortality rate in both the correlation analysis and final model.

## Conclusion

Altitude was a protective factor against COVID-19 mortality. However, the low R^2^ value suggests a nonlinear relationship between covariates and COVID-19 mortality. While this study used linear regression, other studies explore the nonlinear relationship between environmental factors and COVID-19 mortality [7, 59, 60], possibly finding additional associations. Yet, more research is needed in understanding why populations living in different altitudes may have different disease outcomes. Furthermore, in the Ecuador population there seemed to be a protective factor in COVID-19 mortality and identifying as White. Although not statically significant, this finding may be of clinical relevance and help identify areas of improvement in Ecuadorian healthcare.

## Data Availability

The datasets analyzed during the current study are publicly available from the Ecuadorian Ministerio de Salúd Publica national COVID-19 bulletins, https://www.salud.gob.ec/wp-content/uploads/2020/08/INFOGRAFIA-NACIONALCOVID19-COE-NACIONAL-08h00-25082020.pdf and the Ecuadorian Instituto National de Estadìsticas y Censos https://www.ecuadorencifras.gob.ec/estadisticas/.
